# The Effect of Stigma and Social Networks on Role Expectations among African Immigrants Living with HIV

**DOI:** 10.3390/ijerph21060782

**Published:** 2024-06-15

**Authors:** Emmanuel F. Koku

**Affiliations:** Department of Sociology, Drexel University, 3201 Arch Street, Room 288, Philadelphia, PA 19104, USA; emmanuelkoku@drexel.edu

**Keywords:** African immigrants, HIV stigma, social networks, sexual identity, reproductive decisions, role expectations, sub-Saharan Africa

## Abstract

This paper examines how African immigrants living with HIV negotiate and reconstruct their productive (i.e., educational and career opportunities), sexual, and reproductive identities. We used data from a mixed-methods study to explore how stigma and social networks in which participants were embedded shaped how they understood and negotiated their role expectations and responsibilities. Participants revealed how HIV not only changed their identities and limited their sex life, partner choices, and fundamental decisions about fertility and reproduction, but also presented them with the opportunity to reinvent/reshape their lives. Our analysis revealed that the cultural discourses about illness and HIV in participant’s countries of origin, the acculturative and migratory stressors, and the competing influences and expectations from family and friends in their home and host countries shape their illness experience, and how they adjust to life with HIV. This paper builds on sociological understanding of illness experience as a social construct that shapes the ill person’s identity, role, and function in society. Specifically, the paper contributes to discourses on how (i) participants’ social location and identity (as transnational migrants adjusting to acculturative stressors associated with resettlement into a new country), (ii) cultural discourses about illness and HIV in their countries of origin, and (iii) embeddedness in transnational social networks influence health outcomes, including lived experiences with chronic illnesses and stigmatized conditions such as HIV.

## 1. Introduction

Fundamentally, illness is not only a biological condition, but also a socio-cultural phenomenon influencing an ill person’s identity, role, and function in society. A rich line of sociological research has examined illness experience, the social forces shaping this experience, and how the ill individual copes and responds to illness [1,2,3]. Central to this theory is Bury’s classic work “Chronic illness as a biographical disruption” [4], which examined the effect of chronic illness on a person’s identity. Bury conceives of the onset of chronic illness as a disruptive event, fracturing a person’s sense of self and identity, forcing them to either reconstruct a new identity or reinforce existing ones through the mobilization of resources to cope with the disruption.

Other scholars have critiqued, refined, and extended Bury’s original conception of biographical disruption [5], by pointing out that chronic illnesses do not necessarily result in a disruption but the normalization of daily activities and lives. For example, people living with HIV or Ménière’s disease experience shifts between disruption and normalization or what the authors called “biographical oscillation” in their lived experiences [5,6]. Such a process involves the integration of the new illness conditions into their pre-existing lives and selves [7]. Moreover, illnesses do not take place in a vacuum but are influenced and shaped by several processes, notably (a) the ill person’s social demographic background—social class, aging, migratory history, and experience [8,9,10]—and (b) interactions and relationships with others.

Indeed, contemporary work has persistently linked social relationships to health outcomes [11,12,13,14,15,16]. As a social construct, illness experiences shape and are shaped by people’s social networks. The onset of chronic illnesses in particular has been deemed a “network crisis” [17]. According to Pescosolido, such a crisis triggers the mobilization of (external) resources, “bringing individuals, their families, and wider social networks face to face with the character of their relationships in stark form, disrupting normal rules of reciprocity and mutual support” [18]. Thus, social networks have an enduring impact on the lived experiences, orientations, behaviors, and overall responses of the ill person to disruptive events, including living with HIV and other stigmatized conditions [19].

The continuing effort to understand biographical disruption and continuity is even more pertinent to the analysis of lived experiences of people living with HIV (PLWHA). With advances in antiretroviral therapy and treatment [20,21], HIV is now a manageable chronic condition, enabling people to live, renegotiate, and reconstruct their lives, despite the threats posed to their identity and life-chances by the illness [22,23,24,25,26,27]. In particular, how individuals living with chronic illnesses reimagine and renegotiate their lives and role expectations as women/men, spouses/partners, mothers/fathers, and productive members of society can further advance scholarly understanding of illness as a social construct that is subject to the vicissitudes of life course, cultural forces, and social network dynamics.

However, very few studies have examined these role expectations and transitions among those living with HIV, particularly among migrants whose lived experiences are shaped by their location at the intersection of the cultures of their home and host countries as well as the transnational networks in which they are embedded. Consequently, this paper examines (a) how African immigrants living with HIV negotiate and reconstruct their productive, sexual, and reproductive identities, and (b) how stigma and the structure and composition of the social networks in which they are embedded shape this identity negotiation and reconstruction.

## 2. Materials and Methods

### 2.1. Participant and Sample Selection

Data for this paper came from a larger study on HIV stigma and social networks among African immigrants in the Philadelphia region. In recent years, the region has become one of the main destinations of African immigrants, with an estimated population of 40 to 50,000 residing there [28]. More than half came from West African countries, with the largest concentrations from Liberia, Ghana, Nigeria, Sierra Leone, and Togo. African immigrants aged 18–65 years residing in the Philadelphia region were recruited between September 2016 and August 2019 to participate in a study examining the relationship between HIV stigma, social network characteristics, HIV risk, testing, and treatment adherence.

To be eligible for this study, participants had to be residents of the Philadelphia metropolitan region/area; aged 18 to 65 years; born in, self-identify with, or a citizen (at birth) of a sub-Saharan African country; able to read and speak English, French, or other local sub-Saharan African languages spoken within the African immigrant community; and must also be living with HIV. However, participants who were not from a sub-Saharan African region, those who were under 18 years of age, those who were non-residents of the Philadelphia region were excluded from this study.

For purposes of sampling, African immigrants could be considered a “hidden”, “rare”, and “elusive” [29] population partly because of the absence of a true sampling frame for the sub-population, and the documented difficulties in recruiting and engaging them in research [30,31], thereby compounding the challenges of selecting probability samples from the population [32]. Moreover, the practice of aggregating HIV surveillance data on African immigrants with that of African Americans makes it difficult to estimate the overall size and countries of origin of African immigrants living with HIV in many US jurisdictions [33,34,35,36]. Given the above considerations and challenges, this study used a purposive (non-probability) sampling method [37,38] to select participants.

### 2.2. Data Collection, Processing, and Analysis

This study employed a mixed-methods design involving quantitative and qualitative approaches. The quantitative component consisted of a computer-assisted personal interview (CAPI) with 265 respondents (n = 115 HIV-positive; n = 150 HIV-negative/unaware). The qualitative arm consisted of in-depth interviews with 48 participants (n = 20 HIV-positive; n = 23 HIV-negative/status-unaware) and three focus group sessions (one session with 5 HIV-positive participants; two sessions each with 8 participants who were HIV-negative/status unaware). Participants were purposively recruited from community settings (CBOs) and health centers. The primary author administered the CAPI and conducted all interviews and focus groups with the assistance of a trained research assistant and a community health nurse. These interviews and focus group discussions elicited the social meanings and construction of HIV/AIDS, HIV risk, and protective behaviors; the experiences of living with HIV(including responses to stigma, readjustments to life after diagnosis), treatment initiation, and adherence. A qualitative social network inventory was used to collect data on participant’s network members (their socio-demographic characteristics, and their attitudes/behaviors and orientations towards PLWHA), the extent to which they experienced stigma or acceptance from network members, why they concealed/disclosed their status to specific network members, and how treatment and personal decisions related to their life with HIV varied by the characteristics of their social networks. The findings reported here were from the 25 HIV-positive participants who participated in the in-depth interviews and focus groups.

All interviews were audio-recorded and transcribed with any identifying details removed. Two graduate student research assistants (GRAs) checked each transcript for accuracy and, with the assistance of the primary research/author, separately reviewed and coded them [39]. The research team discussed the transcripts and resolved any discrepancies by consensus to enhance inter-coder reliability. To improve the trustworthiness of this study, we employed member checking strategies and external auditing, as recommended by [40]. Selected participants and community members with knowledge and experiences of living with HIV reviewed initial codes and findings and provided further suggestions that helped refine our interpretations. We employed thematic analysis [41] to link emerging themes to research questions and the analytical model. We used the software Dedoose 9.0 for data processing, coding, and analysis [42]. Those who completed this study received USD 35 in compensation in cash/gift cards. Ethical approval for this study was obtained from Drexel University and the Philadelphia Department of Public Health. To preserve anonymity, the names of study participants were replaced with pseudonyms assigned by the primary author/principal investigator.

## 3. Results

### 3.1. Background Characteristics of Participants

Table 1 presents the socio-demographic description of participants. Most were in their mid-30 s (range: 26–55 years) and were either married or in relationship. All (except 4) have children, and close to half (13 out of 25) do not plan to have any more. Almost all participants had attained at least a high school level of education and were also employed, full- or part-time. The average household income (when stated) was USD 30,000 per annum. Most had been in the US for about 10 to 15 years and can speak/understand English, although a few French speakers required interpreters to help them complete the interviews. All participants had families in sub-Saharan Africa, whom they routinely contacted or met in person during periodic visits. Some also had family members in the US and maintained active ties with them and other members of the broader African immigrant community. Almost all participants self-identified as heterosexuals, had been living with HIV for approximately 8 to 10 years, were on antiretroviral therapy, and reported undetectable viral loads.

### 3.2. Context of HIV Diagnosis

Our participants’ experience and responses to HIV diagnosis and stigma need to be contextualized within their lives as transnational migrants. They arrived in the US via many routes: either as students, spouses, legal migrants, refugees, or undocumented. When asked to describe their current life conditions in the US, many were grateful for their success (in attaining education, health, and employment), but also reported varying acculturative stressors relating to poverty, housing, mounting family responsibilities, language barriers, and legal documentation concerns. In addition, African immigrants present with unique health needs: they have a disproportionately higher risk for HIV compared with US-born blacks and face stark barriers in access to HIV prevention, treatment, and other health services than do U.S.-born and other ethnic minorities residing in the country [33,36,43,44,45,46,47].

In the context of these stressors and challenges, the diagnosis of HIV became a major disruption in their migratory journey as they had to adjust to the reality of living with HIV, how/where they became infected, whether/how/to whom to disclose, and the implications of such a diagnosis on their present and future lives. Aside from disruptions associated with and resulting from diagnosis, regular clinic appointments, blood work, and the eventual initiation of antiretroviral therapy emerged as a secondary type of biographical disruption, as it required them to immerse themselves in and alter their schedules to meet treatment demands.

### 3.3. Adjustment to life with HIV: Stigma and Role Alteration

Regardless of the disruption from their diagnosis, interviewees tried to reestablish some sense of normalcy and continuity to their lives, a process that entailed a cognitive review of their current situation—their life goals, plans for family formation, employment, among others. To Bury, individuals tap into cognitive and material resources in their response to biographical disruption [4], including several restraints to cope with the impact of their diagnosis on themselves. Almost of all our respondents changed some salient aspects of their lives and gave up activities that may risk transmission of the virus, while they strived to forge new identities and restore and intensify existing ones through marriage, sexual relationships, parenting, employment, and other social/religious engagements.

#### 3.3.1. African Discourses and Values: HIV, Family Responsibilities

Participants’ responses to biographical disruption must be framed within their identities as Africans (with unique cultural values, beliefs, and practices), as well stigmatizing discourses that shape how they interpret their lived experiences with HIV. To be sure, African immigrants are differentiated by their national, linguistic, cultural, religious, and social backgrounds. Key elements of their culture and belief systems (e.g., social support) are protective from HIV risk, whereas others such as patriarchy, early marriage, fertility preferences, gender inequality, and concurrent sexual partnerships increase their vulnerability to HIV [48,49]. Within these cultures, HIV is framed by four predominant discourses: *mortality*, which sees HIV as a “death sentence”; *immorality*, which associates HIV with (sexual) promiscuity and deviance; *retribution*, which deems HIV as punishment for the contravention of religious edicts; and the discourse of *invisibility*, reflected in the generalized silence and discomfort about HIV as a taboo subject, often referenced with third-person pronouns such as “it”, “thing”, “this”, or “the disease” [49,50,51]. Despite increased HIV knowledge and progress with stigma reduction programs in sub-Saharan Africa, these discourses continue to fuel negative attitudes towards HIV and PLWHA [52,53] in the region, survive through migration, and are reinforced through continuing contact by African immigrants with their countries of origin [48,54].

These discourses fueled stigma towards participants because of either a confirmed or suspected HIV status. The forms of stigma experienced varied, but they manifested mainly in the desire for physical and social distance by participants’ network members and the wider African immigrant community. Some were judged and subjected to reprehensible gossip, while others lost friends and family because of their status. As a result, some participants anticipated stigma from kin and friends, though they were pleased (and, in some cases, pleasantly surprised) by the support they experienced after disclosure or breach of their statuses. All participants were vigilant about when/where/how they took their medications, while some traveled distances to see their HIV care providers and doctors. Their narratives also reflected high levels of internalized stigma, as they all had to cope, at various points, with the shame of having HIV. 

These HIV-related discourses and experiences of stigma also take shape within the broader context of African cultural values and expectations about individual responsibilities to the welfare and sustenance of their families, communities and groups. African women and men are expected to fulfill specific role responsibilities through marriage and procreation to extend their lineage [55,56]. In many patrilineal societies for example, a man is considered a man only if he produces a child (particularly a male) to extend his lineage, while a woman is expected to marry, maintain a home, and bear children as a sign of respect and prestige not only to herself but also to her entire family [57]. In a similar vein, children are expected to undertake the necessary training (formal and informal education, apprenticeship) as they transition to adulthood to care for their own families, parents, and assume the mantle of leadership in their communities [58]. Any threat to the fulfillment of these expectations is a threat to their identity and “face” within their families and broader community networks [59,60]. As a result, participants negotiated their sexual relationships, decisions about whether to have children or not, responsibilities to their spouses and children, and other broader decisions about their lives in the context of these role expectations, while striving to avoid the stigma of living with HIV. One of these decisions relates to sexual relationships.

#### 3.3.2. Negotiating Sexual Relationships

As men and women, sexual identity is one of the most intimate and salient aspects of one’s identity that was not left unscarred by HIV. Participants who were not married or in a relationship at the time of diagnosis faced major challenges in initiating contact or engaging in sexual relationships due to stigma. For many, the decision to enter a relationship had to be weighed against the risk of disclosure and acceptance by their partners. Anella explained her dilemma: “*The last time, the doctor asked me ‘how is your sex life’. I looked at that young man and asked myself, how do I answer?. He asked me 3 times. And he said, I have seen that you don’t answer the question. But then he said he knows how I feel, but that you cannot punish yourself. So he encouraged me to go on a date. I told him I get dates, but I never wanted to go because I am afraid of stigma. I don’t know how I would react if my partner does not accept me*” (Anella, a 46-year-old woman).

While Anella was interested in exploring and having intimate relationships, she was equally concerned about younger generations who were living with HIV and the longer-term impact it would have on their sexual lives: “*It has even made young girls or women not be intimate any longer. When I see the young ones, I wonder how they will live their life. They have to get married, get children. With stigma, this is difficult. How do you do this with a partner and you have to disclose. This woman in the [support] group told me ‘this man is loving me, but I don’t know what to do I am scared of the day I will tell him, I am afraid he will just leave me*”. In light of these restraints, other participants such as Tirida (a 38-year-old woman) became vigilant about when/how to negotiate disclosure, intimacy, and stigma: “*I got this boyfriend, did not want him to get it, this and that, I know. but I told him., oh my goodness, it was very frustrating. he didn’t want to touch anything I touched… Oh my goodness! it took time for him to get it, that we can’t just have sex like sex. I have to protect him*”.

To become involved in or maintain intimate relationships, some participants concealed their status from sexual partners while insisting on consistent condom use. Yaoko (a 47-year-old man), for example, did not disclose his status to his partner, because he did not feel “*close*” enough to reveal the secret, given the context of their relationship*:* “*I am here because I need papers. she marry me. I spend some night here. we get sex, but use condom. first she said no condom, but said i don’t want no more children, so we use it. I don’t tell her [that I have HIV], when I get my papers, is over. like a business*”. For a few, HIV infection signaled infidelity and the impending end of the marriage. Tirida stated: “*my relationship ended when I discovered that I had this (HIV) because I didn’t know that I have it [from him] and when I had it, it made me realize… it made me terminate everything… a relationship that was built on lies*”. Other participants expected their partners to leave them following diagnosis and disclosure because becoming infected with HIV implied moral failure. Lamila (a 41-year-old woman), for instance, was deserted by her husband after diagnosis, although she was still torn between taking “*that risk to meet new people at her age*”, or “*just keep to my husband and convince him to come back and we live together*”.

Those who had or entered new relationships with serodiscordant partners required different role adjustments dictated by the need to protect and prevent exposure to HIV and other sexually transmitted infections. To maintain sexual fulfillment, some adopted several non-penetrative sexual practices, mutual masturbation, and intimate touching. Many, however, had to use condoms out of necessity to protect their partners (and themselves from secondary infections). For Karolina, it was the constraints around sexual intimacy and failure to experience what she defined as “*a real woman*” that depressed her: “*I miss sex…the normal sex, not condom sex, everything; with condom, not the same…like I am not a real woman.*” (Karolina, 26-year-old woman). While some partners understood the necessity to engage in protected sex, others felt it deprived them from the pleasure and intimacy they desired, and sought pleasures elsewhere, usually with other seroconcordant partners, as Kanjobi (a 38-year-old man) explained: “*My woman friend—we meet when my woman travel. we don’t know use condom. she is also married, but I think she also has it. We know we need to satisfy us, she does not like condom, and I also want to enjoy sex*…”.

#### 3.3.3. Reproduction: A Dilemma

Negotiating protected or unprotected sex became even more challenging for those who want children and need to choose between the potential risk of HIV infection or fulfilment of fertility desires, leaving many participants to vacillate between two choices: to either have a child or not.

*Decision to not have a child:* The uncertainties about life with HIV, physical health, and concerns about the potential of transmitting the virus to their partner and unborn child led some participants to decide against having a child, contrary to societal and cultural expectations about reproduction. For instance, Kolifi (a 30-year-old man) has one child and would have loved to have more children, but he was petrified about exposing his wife and any other child to HIV; hence, he had to live what he calls “if only’s” (regrets) over unfulfilled fertility desires. BoJonso (a 31-year-old woman) expressed fears of vertical transmission: “*Man, you know this stuff, you can give it…, I did not want to have any more kids—I put myself on birth control I don’t want to put anyone in trouble”.* To Mansana (a 33-year-old woman), the dilemma was almost heartbreaking: “*I feel my HIV be is… big problem. The doctors tell my husband that we use condom. He like… like want children bad, and I not want him to get it. He want to do without condom. I refused then he start to use it*”. For BoJonso and Mansana, the availability of pre-exposure prophylaxis (PrEP) was a welcome option that could allow them to fulfill their fertility and sexual desires, while reducing the risk of HIV infection. But many participants were not aware of PrEP, and the few who did held inaccurate perceptions about its use and efficacy. Both BoJonso and Mansana discussed PrEP with their doctors; however, BoJonso was not willing to encourage her long-term boyfriend to use it, due to concerns about risk exposure: “*Yes, we discussed it with the doctor, but I can’t ask him to take it…maybe if he wants to take it. I think he is not interested…last time, he said he is ok, and doesn’t want to discuss it. I also worry. If he forget to take it and get HIV and sick … so no more baby… we use condom*”. Mansana, on the other hand, was interested in and discussed PrEP with her husband extensively: “*Since he not like condom, I talk to doctor. She talk PrEP… to me and him* [my husband]. *But he refuse…say he cannot take medicine everyday… but we talk about it again … maybe he change mind*”.

Other participants such as Punda were not sure if anyone would accept or love them enough to have kids with them, so she decided not to have children: “*I mean due to the HIV, I don’t know how it will work. hmm, you just feel like you know you can’t, in my case I just feel like I can’t have any friend any type of friend, relationship with anybody …—nothing is working for you, you’re not gonna have kids- that’s why I said I don’t even know if I will. So, there’s so many things… cause who’s gonna go or like want to have sex with you if you have HIV and give you kids, you know? If you don’t have money, you can’t go to doctor to help have babies… Yeah, yeah you can do it but if you don’t have money, no*” (Punda, a 34-year-old woman).

*Decision to have a child*: However, due to cultural considerations, other participants decided to have children, as a claim to normality and to deflect blame or stigma. In African contexts, certain identities such as that of a mother or father are so salient and culturally ingrained that a decision to not have a child raises suspicions about one’s virility, fertility, and status as a respected member of the society. Thus, for some women and men living with HIV, having a child may be a way to avoid unwanted attention, mask, or divert attention from its related stigma [61,62]. For these participants, the prestige of continuing their family lineage, against the inherent stigma against infertility, was a strong motivator for having their own children, despite the fear of transmission. After years of refusal to get married or have children, Banito (a 41-year-old man) decided to throw in the towel. When probed, he explained: “*I need children, but I not trust if the woman can get it too. I was fearing… Mami [Mother] give me hard hard time, many questions she ask. I tell her I go to school, no time. One day she ask if I be a man, all ok ‘down there’. I know I have to prove. If I no marry, get children, they think I like men* [as gay]*. With child, no question, I am man. I call him son… all ok… my brother!. Doctor* [Name withheld] *help big time*”.

As alluded to by Banito, almost all participants who were contemplating or concerned about having children discussed their plans with their providers and explored alternative reproductive options. Some participants such as Punda noted that they seriously considered in vitro fertilization (IVF) but were deterred by its cost, while others such as Erikus were grateful for partners who were willing to go on PrEP: “*I lucky… my woman is good…we want many babies* [laughs]*, but I get HIV. She ok…no HIV, negative, nshalaa… we see doctor, she take medicine… we take together… so that help we get child now… nshalaa… she good woman…*”. For a few other respondents, the desire for children became so great that they separated from their existing discordant relationships (where they believed it was not possible to do so safely) in search for new partners.

For others, the need to secure the relationship with their partners was of paramount concern, as explained by Lila (a 31-year-old woman): “*Like my last child, husband forced that we have baby… he said he didn’t care, and I want him to be for me, so we have her*”. Kanji (a 36-year-old woman) clarified the frustrations she was experiencing with her husband (who was HIV-negative) and his desire for more children. “*When we start, we said we get one child, it was ok, hmmm? He get one before we start, so ok? We get child, everyone happy, like we plan. Now he say he want one baby, every day, he say we get one more. I scare for baby that I give it to him too. Now he angry. I scare he get another woman*”.

Gay participants felt pressured to get married and conform to cultural expectations of masculinity and parenthood. Henrik (a 33-year-old gay man), for example, resisted repeated attempts by his family to set him up with a woman. To keep their families happy and be “*in-line*” with cultural expectations, some gay/bisexual PLWHA facing such pressures hid their status from family and potential partners, got married, lived on the “down-low”, and had children. Explaining the basis of the pressure further, Henrik observed: “*our community is so steep!…I have a friend of mine, he’s gay from [country-blinded] but he has to get married to a woman because he will never tell nobody that he like men or something like that… They will kill him.*”

#### 3.3.4. As a Parent (for Those with Children)

For participants with children, disclosure was an indirect indicator of past experiences and decisions they would rather want to keep secret from their children/family. Kabini (a 44-year-old man) was conflicted about if/when to disclose to his son: “what can I tell my boy one day …that Daddy was f**ing and not use condom…when I advise him do this and don’t do that?”. However, the degree of ambivalence felt by these participants depended on the degree of culpability they had for infection. While most of the men interviewed blamed themselves and (in a few cases their partners) for their risky sexual choices, the women on the other hand felt victimized by unfaithful partners and used disclosure to their children to explain and teach them lifelong lessons about sexual fidelity.

Ultimately, for most participants, disclosure/concealment became a duty to care and protect themselves and their kids from stigma and risky behaviors. To Susula, disclosure is important but timing matters: “*for me it is not good to hide it. if you hide it, you can be yourself. It is important for some people to know at the right time. Now I am 55. If God permits and I am 65, 70, I will call my children and tell them you know I have this for previous days. But I didn’t tell you before because I don’t know how you are going to react or how you feeling. so I have to tell you to protect yourself*” (Susula, a 55-year-old woman).

On the contrary, Erikus believed that it was not necessary to disclose. So long as he was in good health, it was better for his children to remain in the dark for their and his own peace of mind: “*I know I have to tell them…but it’s not important, maybe one day. But I am doing good. I have good health, not sick. So, it’s just like any other disease. If I tell them, they will think I am going to die. They will be worried about me. And I will be worried they will tell someone…so, I keep it to myself…better for me, better for them*” (Erikus, a 33-year-old man).

#### 3.3.5. Productive Responsibilities

All participants reframed HIV diagnosis and the availability of treatment as a chance to reinvent their lives and give back to society. Mounting responsibilities from extended families abroad exerted added stressors on participants’ adjustment and integration, demanding more of their attention. Fatubo (a 38-year-old woman) observed that: *“I have children, three children. Two girls, one boy. I taking care of first daughter [who] came here with husband and I living with them, taking care of their children… HIV is a big problem, but these bigger. HIV not the problem here”.* As a result, many (re)-engaged in other productive activities (such as taking up education and other career opportunities) to fulfil family expectations and obligations, support extended family members within and outside the United States, and increase their social mobility while adjusting to life with HIV.

### 3.4. Effect of Social Network and Cultural Contingencies

These adjustments to “biographical disruptions” caused by HIV depend largely on the structure and composition of participants’ transnational networks. In particular, a person’s involvement and integration in community activities (as evidenced by regular attendance and visibility) increase their exposure to prevailing norms and attitudes. By the same token, integration into a transnational network of ties increases participants’ awareness of cultural expectations around family lineage and procreation, an awareness that is reinforced by frequent contact with family members in their countries of origin. For participants such as Priestus (a 48-year-old man), the pressure to meet these expectations became so intense that he relented: “*my girlfriend here (negative will not have more children). I went home, my aunt arranged a girl for me, and we have a child …both my woman and my child are home and safe*”.

Anelaka (a 31-year-old woman) went further: “*my mother wants grandchildren …she kept pressuring me. When I said I am not ready, she gets angry, and tries to get other people involved in my business, gives my number to men… eventually …you can’t keep saying no to men…I pray for a good man” For these participants, as Beyeza-Kashesya aptly summarized: “The moral imperative may be to conform to the norms or risk being categorised as abnormal among one’s social network.*” [63], as Priestus and Anelaka did.

Aside from integration, the composition of participants’ networks (the knowledge and attitude of their network’s members) has an important bearing on how they interpret and make sense of their cultural expectations, experiences of stigma, and its resultant role strain. Henrik has a network comprising people from different backgrounds, who accepted him without stigma, and exposed him to new and alternative means of resisting the pressure from his parents. Though his parents were not satisfied with his decision to not have children, he felt comfortable given the support he experienced from others in his network. Elaborating on the benefits of the diversity of his network, he explained:

“*Look on this list. oyibos* [slang for whites]*: HR28 and HR30 Whites; HR31, my girl from China; HR33 Indian; HR45 from South Africa—all from my work; they are good. They are different, they have no connection with my [African] people in the city, so I now relax, no rumors, gossips. We talk about HIV, they get tested too. Some of them know I have it [laughs]. I told them—HR45, HR31, HR28. some told me they have it. I know the stigma is here, but I don’t feel it. You learn a lot more. But none of my African friends knows this. I only worry when I go shopping in the city. feel someone is looking at me… you know. Maybe is me. But I still have to be careful about who I hang around. Don’t get me wrong. I love my people, I miss my community.… But being far away gives me options—who to invite, visit. I choose*” (Henrik, a 33-year-old gay man).

The fact that Henrik was not deeply integrated within his African network helped shield him from normative expectations regarding family formation. For others such as Anelaka, access to other participants who were similarly living with HIV moderated their experiences and provided them with alternative means of adjusting to their roles. She explained that the other PLWHA she met through an online support group provided her with a list of online dating sites for PLWHA, increasing her access to potential dates. To her, these homophilous ties (those who shared the same HIV status with her) were instrumental in helping ease her adjustment after diagnosis, and could also help her meet a suitable partner and start a family.

## 4. Discussion

This paper used data from a larger mixed-methods study to examine (1) how African immigrants living with HIV negotiated and reconstructed their productive, sexual, and reproductive identities, and (2) how stigma and the structure and composition of the social networks in which they are embedded shape this identity negotiation and reconstruction. We showed that participants’ experience and responses to HIV diagnosis and stigma were shaped by their lives as transnational migrants. While some had increased their social mobility and educational attainment, others described their lives as characterized by precarity—unemployment/poverty, housing, mounting family responsibilities, language barriers, and legal documentation concerns—and other acculturative stressors.

In these contexts, diagnosis with HIV and the subsequent insertion into and initiation of treatment became a major disruption in their migratory journey as they had to adjust to the reality of living with HIV. Regardless of the disruption from their diagnosis, interviewees tried to reestablish some sense of normalcy and continuity to their lives, a process that entailed a cognitive review of their current situation—their life goals, plans for family formation, employment, among others. HIV not only affected them physically, in terms of their adjustments to the side-effects of their mediations, but it also shaped their sense of self—how they view themselves vis à vis others. These identity reappraisals were usually filtered through interactions with their social network ties, some of whom affirmed their identities (with support) while others stigmatized them.

In particular, participants observed that HIV has changed their self-identity and limited their sex life, partner choices, and fundamental decisions about fertility/reproduction in ways that were resonant with those observed among other groups [64,65,66,67,68,69]. For some PLWHA, having to come to and accept a decision to not have children was a rational response to (internalized) stigma, shame, and guilt about their reproductive potential, despite the promises of assisted and safer means of reproduction. For others, the decision to get married and/or have children was a strategic means of concealing their HIV status and/or deflecting attention from it [62]. As a form of biographical reinforcement [22], they were a means of maintaining their standing (or face) in the community and a source of renewed self-worth, conformity to social expectations around childbearing, and the continuity and reinforcement of parental identities held or expected before the disruption by HIV illness. For example, research has shown that women living with HIV often privilege and stress the primary importance of meeting their responsibilities as mothers and wives, regardless of the challenges posed by their HIV status, as some of our participants pointed out. They saw the disruption caused by an illness as an opportunity for biographical reinvention, and drew on resources, resilience, and other assets to transition to and maintain their new identities as non-smokers [70] or to reinvent their sense of self and future after the diagnosis of HIV [71].

To many partnered participants, HIV infection was the elephant in the room, the silent third-party that became more visible whenever their HIV-negative partner appeared to be seriously sick. The ever-present specter of risk increased their vigilance about the accidental exposure/transmission of HIV. At the same time, these adjustments were also a source of strength to many participants and demonstrated their love and support for each other and other network members (such as parents, children, intimate partners). Those who remained in their relationships became more bonded over their shared desire to stay together and take care of their children or each other, regardless of their HIV status. A critical resource that participants leveraged was their social capital. While they recognized the social support available in their immigrant communities, they were also aware that these very communities reinforced stigmatizing discourses as well as normative expectations around family roles and responsibilities, and exerted pressure on PLWHA to come up with explanations about their role responsibilities and decisions, without disclosing their HIV status. To counter the stigma and expose themselves to countervailing norms, many PLWHA forged ties with network members outside the African immigrant community, including others living with HIV, for commiseration and further support. Almost all participants also turned or held to their religious faiths to cope with these changes and adjustments.

As a result of these challenges with reproductive choices and decisions about sexual intimacy, newer reproductive and HIV prevention technologies offered hope to PLWHA to meet their needs. Many participants have heard of the feasibility of assisted reproductive technologies (such as sperm washing, in vitro fertilization) but noted their inability to afford them. Other participants have also heard that PrEP (pre-exposure prophylaxis) and PEP (post-exposure prophylaxis) may offer new hopes for preventing HIV, especially among those who are in serodiscordant or other sexually risky relationships [72,73], but have little understanding of how it works, its availability, or how to overcome the stigma and other misconceptions surrounding its use [74,75,76]. Some participants pointed out that their doctors and other professionals became partners in helping them conceive and have children safely. In this vein, it is imperative to continue to provide education, information, and resources that address the reproductive and sexual needs of PLWHA in a culturally informed and respectful and receptive manner, using providers of appropriate age and gender, given the sensitivity of sex as a topic in African (immigrant) cultures [77,78].

## 5. Limitations and Strengths

There were limitations to this study, relating mainly to its design and recruitment of participants. This study used cross-sectional data collected purposively from a small sub-sample of African immigrants in the US. Thus, our findings may not be representative of the experiences of many other African immigrants in the country. Additionally, because we did not collect data from participants over time, we are unable to explore how their responses to role expectations and their reproductive desires changed with the entry and exit of friends, associates, and family members from their personal networks over their illness trajectory. However, some of our findings align with those reported in other studies on African immigrants and other groups [79,80,81,82,83]. Finally, given the sensitivity of HIV as a research subject, some community members were reluctant to participate in this study owing partly to documented concerns among African immigrants about participating in clinical studies [30,31]. Thus, we adopted a recruitment strategy (i.e., purposive sampling) to successfully reach participants in both community and clinical settings. However, participants recruited from clinical settings were likely those engaged in care, with different expectations and experiences of adjustment to life with HIV, introducing a possible recruitment bias.

Despite these limitations, our analysis contributes to theoretical work on biographical disruption and repair by showing that participants’ responses and adjustments to life with HIV are shaped by experiences of stigma within their social networks. According to Pescosolido and Manago, stigma and its sequelae are “fundamentally tied to, negotiated in, and given meaning through the [crucible of] networked social interactions” [19]. An individual’s social network—whom they know, whom they talk to and how often, and whom they confide in or disclose their HIV status to—are critical to shaping their adjustment to life with HIV and fulfillment of their role expectations and reproductive/productive desires.

Since 2019 when we concluded data collection for this project, the epidemiology of HIV has changed, with the wider availability of ARV(antiretroviral) medications and other treatment options, PrEP (pre-exposure prophylaxis), and PEP (post-exposure prophylaxis), leading to the gradual routinization of “treatment as prevention” or “undetectable = untransmittable” protocols [84]. These advances have increased the life expectancy of those living with HIV and their potential to live full and fulfilled lives, yet we know very little about how they have shaped the experiences and responses of PLWHA. As a result, it is imperative to collect more detailed data, using longitudinal and other appropriate designs, to illuminate how PLWHA adjust to the realities of living with a chronic condition, over their life course, in the context of changes in the epidemiology, treatment, and societal attitudes to HIV.

Our findings suggest the need for more support services for those living with HIV as they reconstruct their lives following diagnosis. Such services must include education about and the navigation to reproductive, sexual, and mental health resources to help participants cope with the challenges of living with HIV and attain and lead full productive lives. It is imperative to deliver these educational and related interventions in a culturally informed and respectful and receptive manner, using providers and health navigators of appropriate age and gender [85]. Above all, stigma reduction efforts must continue to stress the “normalcy” of HIV as a chronic condition to ensure wider accepting attitudes from the general public and participant’s personal networks.

## 6. Conclusions

This paper explored how African immigrants living with HIV negotiated and ‘repaired’ the disruptions to their identities and role expectations and functions. Our analysis builds on and contributes to research that sees illness as a socio-cultural construct that not only disrupts an ill person’s biography but also influences their identity and transforms their role expectations as women/men, spouses/partners, mothers/fathers, and productive members of society. These adjustments must be understood in the context of the ill person’s cultural background and social location. For transnational migrants especially, the cultural discourses about illness and HIV in their countries of origin, acculturative stressors associated with their resettlement into a new country, and the competing influences and expectations from transnational social networks they maintain in their home and host countries differentiate and shape their illness trajectory, experience, and how they adjust to and negotiate the reality of life with HIV.

This paper contributes to the theoretical and scholarly discourse on how migrants’ social location, identity, and embeddedness in transnational social networks influence health outcomes, including lived experiences with stigmatized illnesses such as HIV.

## Figures and Tables

**Table 1:** Socio-demographic characteristics of HIV-positive participants (N = 25).

	Men	Women	Total
**Number of Participants**	**10**	**15**	**25**
**Region of Birth**			
Southern Africa	0	3	3
West Africa	5	8	13
Central Africa	3	2	5
East Africa	2	2	4
**Sexual Orientation**			
Heterosexual (Straight)	8	15	23
Homosexual (Gay)	2	0	2
**Number of Children**			
No Children	1	3	4
1 Child	6	4	10
2 to 3 Children	2	5	7
4+ Children	1	3	4
**Desire for More Children?**			
Yes	6	2	8
No	2	11	13
Maybe	2	2	4
**Marital Status**			
Married/Domestic Partnership	4	7	11
Divorced/Separated/Widowed	2	3	5
Single (Never Married)	4	5	9
**Highest Level of Education**			
No Schooling Completed	1	1	2
Primary/High School	3	2	5
Some College/Associate Degree	3	7	10
Bachelor’s Degree	1	4	5
Post-Graduate Degree	2	1	3
**Employment Status**			
Employed-Full/Part Time	4	8	12
Self-Employed	1	4	5
Not Employed	5	3	8
**Religious Affiliation**			
Protestant	2	6	8
Christian/Pentecostal	2	5	7
Catholic	1	2	3
Muslim	4	2	6
Traditional/No Affiliation	1	0	1
**Age (Mean)**	40	35	37.2
**Years Living in USA (Mean)**	11	10	10.5
**Years Living with HIV(Mean)**	6	8	7.5

## Data Availability

The data presented in this study are available on request from the corresponding author.
